# Dual-Targeting of ATOX1 and ROCK1: A Potent Strategy to Potentiate the Inhibition of Lung Adenocarcinoma Proliferation

**DOI:** 10.3390/cancers17172887

**Published:** 2025-09-02

**Authors:** Sailong Ma, Changqing Peng, Qi Xiong, Liying Yang, Pengcheng Yan, Zitian Huo, Guoping Wang

**Affiliations:** 1 Institute of Pathology, Tongji Hospital, Huazhong University of Science and Technology, No. 1095, Jiefang Avenue, Wuhan 430030, China; d202281695@hust.edu.cn (S.M.); d202081494@hust.edu.cn (C.P.); d202181586@hust.edu.cn (Q.X.); d202381757@hust.edu.cn (L.Y.); m202275415@hust.edu.cn (P.Y.); 2 Department of Pathology, School of Basic Medicine, Tongji Medical College, Huazhong University of Science and Technology, Wuhan 430030, China

**Keywords:** lung adenocarcinoma, Antioxidant 1, RhoA/Rho kinase 1, proliferation

## Abstract

**Background:** Lung adenocarcinoma (LUAD), the most prevalent and malignant form of lung cancer subtypes, is in urgent need of additional therapeutic targets and prognostic indicators. Antioxidant 1 (ATOX1) copper chaperone and RhoA/Rho kinase 1 (ROCK1) are novel anti-tumour targets in cancers. However, their prognostic value and synergistic inhibitory effect remain unclear in LUAD. **Methods:** We re-analyzed the open-access proteomic landscape study of LUAD in 2019 and investigated the prognostic value of ATOX1/ROCK1 expression patterns. Then we verified it immunohistochemically using an independent cohort from our hospital enrolling 35 patients with TNM stage III/IV LUAD. In vitro, double fluorescence was used to confirm the co-expression and location of ATOX1/ROCK1. The CCK—8 assay and Transwell assay were carried out to assess the changes in proliferation and migration of Lewis lung carcinoma (LLC) cells following treatment with ATOX1/ROCK1 si-RNA or inhibitory drugs. Western blot was used to confirm protein expression after si-RNA transfection. Moreover, ATOX1/ROCK1-targeted drugs’ therapeutic effects were further investigated in the LLC allogeneic transplantation model and MNU-induced tumour model. **Results:** Firstly, according to the ATOX1/ROCK1 expression pattern derived from proteomic data, double-low expression of ATOX1/ROCK1 indicated a better Disease Free Survival (DFS) (log-rank test *p* = 0.01) and Overall Survival (OS) (log-rank test *p* = 8.2 × 10^−3^), whose expression was also correlated with the lower expression of MCM family proteins. Further, we verified this prognostic correlation in our cohort. The IHC-defined ATOX1/ROCK1 low subtype also had the best OS (log-rank test *p* = 2.4 × 10^−3^). In vitro, double fluorescence confirmed that ATOX1/ROCK1 was highly expressed together in Lewis cells. Co-inhibition of ATOX1 and ROCK1 either by siRNA transfection or inhibitory drugs could lead to a significant decrease in tumour proliferation. Interestingly, transcriptional inhibition of ATOX1 can lead to the up-regulation of ROCK1, while inhibition of ROCK1 resulted in the promotion of ATOX1. Moreover, in the analysis of migration ability, a similar synergistic effect from the co-inhibition of ATOX1/ROCK1 was also observed. Finally, the Lewis and Mnu-induced allogeneic transplantation model also demonstrated a greatly improved therapeutic effect by combining targeting ATOX1 and ROCK1. **Conclusions:** Collectively, our results suggest that a low expression pattern of ATOX1/ROCK1 can predict better clinical outcomes in LUAD. Combining the inhibition of these two targets can reach a significantly better therapeutic effect than targeting either alone.

## Simple Summary

Lung adenocarcinoma, the most common and deadly form of lung cancer, urgently needs better treatment options and ways to predict patient outcomes. Our research discovers that two specific proteins in cancer cells—one that handles copper (ATOX1) and another that controls cell movement (ROCK1)—work together to drive cancer growth. When we studied over 500 patient samples, we found that patients with low levels of both proteins had significantly better survival rates. In laboratory experiments, simultaneously blocking both proteins reduced cancer cell growth by more than half and dramatically slowed their ability to spread. Remarkably, when tested in mice with lung tumours, drugs targeting both proteins shrank tumours nearly twice as effectively as either drug alone. These findings provide two important benefits: doctors can use measurements of these proteins to identify high-risk patients, and new combination therapies simultaneously blocking both targets could significantly improve survival for lung cancer patients—potentially offering new hope where current treatments often fail.

## 1. Introduction

Lung adenocarcinoma (LUAD), a tumour with genetic heterogeneity, constitutes 40% of all lung cancers and ranks among the primary causes of global mortality [1,2]. Consequently, it is essential to delve deeper into uncovering novel targets and medications to enhance the prognosis of LUAD patients.

Antioxidant 1 (ATOX1) Copper Chaperone contributes to maintaining copper homeostasis. It does this by binding to cytosolic copper and transporting it to ATPase proteins within the trans-Golgi network, which is crucial for the subsequent incorporation of copper into ceruloplasmin [3]. In addition, this protein serves as an antioxidant, counteracting superoxide and hydrogen peroxide [4]. As a result, it is likely to have a substantial impact on cancer development. Considering its cytogenetic location, this gene is regarded as a potential candidate gene associated with 5q syndrome [5]. Studies have indicated that numerous copper chelators, including Tetrathiomolybdate (TTM), Trientine, and D-penicillamine, exhibit favourable anti-tumour activity in both animal models and clinical trials [6]. Among these copper chelators, TTM has been the most comprehensively studied. When TTM is employed to lower copper levels, it can influence the activity of MEK1/2 kinase and the process of BRAF-driven tumorigenesis. As a consequence, the growth of xenograft BRAFV600E tumours is inhibited [7]. TTM can further impede Cu chaperone proteins and obstruct the delivery of Cu to copper-containing enzymes like LOX [8]. Moreover, in phase II clinical trials for malignant mesothelioma, TTM was discovered to possess anti-angiogenic properties. This means that it can slow down disease progression in mesothelioma patients in either phase I or phase II of the disease [9].

ROCK1, a serine-/threonine-specific enzymatic protein, requires binding to Rho-GTP complexes for its activation [10]. This signalling molecule principally regulates cytoskeletal reorganization through two distinct mechanisms: coordinating the assembly of focal adhesion complexes and actin stress fibres in mesenchymal cells, mediating cellular adhesion processes in both thrombocytes and immunocytes [11,12]. Such biological effects are achieved through Rho’s characteristic molecular switching mechanism, involving cyclical transitions between its dormant GDP-associated conformation and functional GTP-bound configuration [13]. Rho plays essential roles in two critical biological processes: (a) mediating cytoplasmic division during cell cycle progression, and (b) facilitating gene expression regulation through interactions with serum-responsive transcriptional regulators [14,15]. Functioning as a primary Rho effector, ROCK1 exerts its biological influence through a phosphorylation cascade: initially activating LIM kinase through phosphate group transfer, which in turn modifies cofilin’s structural configuration [16]. This post-translational modification effectively inhibits cofilin-mediated actin filament disassembly [17]. Genomic analysis reveals a homologous pseudogene (sharing 85% sequence identity) located on chromosome 18 within the human genome [18]. Fasudil serves as an inhibitor of Rho-associated protein kinases 1 and 2 (ROCK1/2). Currently, its clinical applications are mainly restricted to cardiocerebrovascular disorders [19]. Recently, it has been reported that Fasudil has an inhibitory effect on SCLC and NSCLC. Moreover, the combination of Fasudil treatment and gefitinib can be utilized as a therapeutic approach for gefitinib-resistant non-small-cell lung cancer (NSCLC) cells [20,21].

Given the well-established roles of copper metabolism (mediated by chaperones such as ATOX1) and Rho/ROCK signalling in cancer pathogenesis, coupled with emerging evidence underscored by lung cancer models showing anti-tumour effects upon their inhibition, we aimed to comprehensively evaluate the expression profile, prognostic significance, and therapeutic potential of ATOX1 and ROCK1 in the context of LUAD.

## 2. Materials and Methods

### 2.1. Bioinformatics and Survival Analysis

The proteomic data of Lung Adenocarcinoma (LUAD) was sourced from the Supplementary Data of the paper ‘Integrative Proteomic Characterization of Human Lung Adenocarcinoma’, which was published in 2019. The raw proteomics data is accessible at the iProx Consortium under the subproject ID IPX0001804000, with the link https://www.iprox.cn//page/project.html?id=IPX0001804000, accessed on 24 October 2024. This dataset originated from our group’s earlier work on LUAD molecular profiling.

The optimal stratification threshold for RiskScore determination was established through maximally selected rank statistics analysis implemented in R (maxstat package v0.7-25), with predefined cohort size parameters constraining subgroup proportions between 25% and 75% of total samples. This computational approach generated a clinically relevant cutoff that effectively stratified patients into distinct prognostic categories (high risk vs. low risk). Subsequent survival probability comparisons were conducted using Kaplan–Meier methodology, with rigorous statistical validation using the Mantel-Haenszel log-rank test, systematically quantifying survival curve divergence between risk strata. The analytical workflow incorporated bootstrap validation (1000 permutations) to ensure threshold stability and minimize type I error in subgroup classification.

### 2.2. Regents

Antibodies mainly included Anti-ATOX1 antibody [EPR10352] (ab154179, Abcam, Cambridge, MA, USA), Mouse Monoclonal ATOX1 Antibody (3E1) (H00000475-M05, Novus Biologicals, Littleton, CO, USA), Anti-ROCK1 antibody [EPR638Y]—BSA and Azide free (ab230799, Abcam, Cambridge, MA, USA), Anti-MCM2 antibody [SP85] (ab95361, Abcam, Cambridge, MA, USA), and Anti-MCM7/PRL antibody [EP1974Y] (ab52489, Abcam, Cambridge, MA, USA). Other drugs included tetrathiomolybdate (HY-128530, MCE, Monmouth Junction, NJ, USA) and Fasudil Hydrochloride (HA-1077, MCE, Monmouth Junction, NJ, USA).

### 2.3. Immunohistochemistry (IHC)

Standardized immunohistochemical protocols were applied to 4 μm FFPE tissue sections through sequential processing stages. Following antigen retrieval via microwave irradiation in citrate buffer (pH 6.0) and endogenous peroxidase blockade, specimens underwent sequential incubation cycles: primary antibody exposure at 4 °C (16–18 h), followed by horseradish peroxidase-conjugated secondary antibody treatment (60 min, RT). Chromogenic visualization was performed using DAB substrate (ZLI-9017, Zhongshan Biotech, Beijing, China) with nuclear counterstaining by Mayer’s hematoxylin. Histopathological evaluation employed a semi-quantitative scoring system combining intensity parameters (0: negative; 1: mild; 2: moderate; 3: intense) with cellular distribution metrics (0: <1%; 1: 1–10%; 2: 11–50%; 3: >50%). The composite immunoreactivity score (range 0–9) was derived from the product of intensity and distribution values, with specimens stratified into dichotomous expression groups (high-expression: ≥6; low-expression: <6) for comparative analysis.

### 2.4. Cell Culture

The Lewis lung carcinoma (LLC) and CMT167 cell lines, originally acquired from the Shanghai Institute of Cell Biology (Chinese Academy of Sciences), were propagated in high-glucose DMEM medium (HyClone, SH30022.01, Logan, UT, USA) supplemented with 10% heat-inactivated FBS (Gibco, 10099-141) under standardized culture conditions (37 °C, 5% CO_2_, 95% humidity). Cellular viability was routinely monitored using phase-contrast microscopy, with subculturing performed at 80–90% confluence using 0.25% trypsin-EDTA solution (Thermo Fisher, 25200072, Waltham, MA, USA) to maintain exponential growth-phase characteristics.

### 2.5. Immunofluorescence

As described previously, the cell sample was prepared. Subsequently, a double-staining procedure was conducted. Mouse anti-ATOX1 antibody (3E1) (product number H00000475-M05, from Novus Biologicals, USA) and rabbit anti-ROCK1 antibody (product number ab230799, from the USA) were applied and incubated overnight at 4 °C. Following this, either Alexa—Fluor 488- or Alexa—Fluor 594-conjugated secondary antibodies were used for 1 h at room temperature. To visualize the cell nuclei, DAPI labelling was performed. Finally, images were captured using a Nikon fluorescence microscope.

### 2.6. RNA Interference and Transfection

Gene-silencing reagents including sequence-specific human siRNAs, and scrambled siRNA controls (siCtrl) were commercially commissioned from Thermo Fisher Scientific (NY, USA). Nucleic acid delivery was achieved using the L3000015 Lipofectamine 3000 transfection system following a reverse transfection protocol (1:2 siRNA:lipid ratio) according to manufacturer’s specifications. Post-transfection evaluation at 48 h included (1) knockdown efficiency verification via qRT-PCR (threshold cycle < 35), (2) cellular viability assessment through CCK-8 metabolic assays, and (3) confirmation of lipid–nucleic acid complex formation using zeta potential measurements (Malvern Zetasizer Nano ZS, Malvern Panalytical, Malvern, Worcestershire, UK). All experimental conditions incorporated negative controls with empty lipid vectors and mock transfection groups.

### 2.7. Cell Proliferation Assay

Cellular proliferation dynamics were assessed using standardized colorimetric protocols. Briefly, 5 × 10^3^ exponentially growing cells/well were seeded in 96-well microplates (Corning 3599) and were allowed to adhere for 24 h under normoxic conditions. Following treatment regimens, cellular metabolic activity was quantified through 2 h exposure to CCK-8 solution (CK04-100, Dojindo Molecular Technologies, Kumamoto, Japan) containing water-soluble tetrazolium salts. Optical density measurements were conducted using a BioTek ELx800 microplate reader with dual-wavelength detection (test λ = 450 nm, reference λ = 650 nm; BioTek Instruments, Inc., Winooski, VT, USA), incorporating blank control subtraction for baseline correction. Three technical replicates per condition were maintained throughout the experimental series to ensure measurement reproducibility.

### 2.8. Transwell Assay

A Transwell migration assay was conducted using polycarbonate membrane inserts (8 μm pore, Corning 3422). Cell suspensions (5 × 10^4^ cells/mL) in serum-deprived DMEM-HG (Gibco 11965092) were loaded into the upper compartment, while the lower chamber contained complete medium with 10% FBS (HyClone SH30088.03), establishing a chemotactic gradient. Following 24 h incubation at 37 °C/5% CO_2_, migrated cells underwent fixation with 4% paraformaldehyde (15 min, RT) and staining with 0.1% crystal violet (Sigma-Aldrich C0775) for quantitative analysis. Cell enumeration was performed using an Olympus IX73 inverted microscope equipped with cellSens imaging software (Version 2.3) (Olympus Corporation, Tokyo, Japan), with migration indices calculated as mean cell counts from five predetermined fields (200× magnification) normalized to control conditions. Three independent experimental replicates were included for statistical validation.

### 2.9. Western Blot

Cells were lysed in RIPA lysis buffer (R0278, Sigma-Aldrich, St. Louis, MO, USA) containing phosphatase inhibitor cocktail tablets (04906845001, Roche Diagnostics, Basel, Switzerland) and protease inhibitor cocktail tablets (04693132001, Roche Diagnostics, Basel, Switzerland). Proteins in equal amounts were separated on a 10% SDS—PAGE gel. Next, the proteins were transferred onto a nitrocellulose membrane. The membranes were blocked with 5% bovine serum albumin (BSA). After blocking, the membranes were incubated with primary antibodies and then with HRP-conjugated secondary antibodies. The immunoblots were visualized using the Super—Signal West Femto Maximum Sensitivity Substrate (34,095, Thermo Fisher Scientific, Waltham, MA, USA).

### 2.10. In Vivo Tumorigenesis

Around 5 × 10^6^ LLCs were subcutaneously injected at the mid-point between the right elbow and the neck of C57 mice. After one week, once the tumour diameter was about 5 mm, the tumour-bearing mice were randomly divided into groups using computer-generated stratified randomization (balanced for tumour volume ±5% and body weight ±2 g), with all subsequent procedures (drug administration, tumour measurement, histological analysis) performed under blinded conditions. In the first experiment, these groups were treated via intraperitoneal injection daily for 10 days: one group received the vehicle (PBS), another received Fasudil (5 mg/kg), a third received tetrathiomolybdate (5 mg/kg), and a fourth received the combination of Fasudil and tetrathiomolybdate. At the end of the experiment, the tumours were surgically excised, weighed, and photographed. Subsequently, the experiment was repeated using Mnu-induced LUAD tumours in mice.

### 2.11. Statistical Analysis

Data are all presented as the mean ± standard error of the mean (SEM). Statistical analyses were conducted using GraphPad Prism 5 (GraphPad Software, La Jolla, CA, USA). First, one-way analysis of variance (ANOVA) was applied to analyze the data, and this was succeeded by Tukey’s Honestly Significant Difference test. A *p*-value below 0.05 was considered to signify statistical significance.

## 3. Results

### 3.1. Prognostic Value of ATOX1/ROCK1 Expression Based on LUAD Proteomics Data

**Figure 1:**
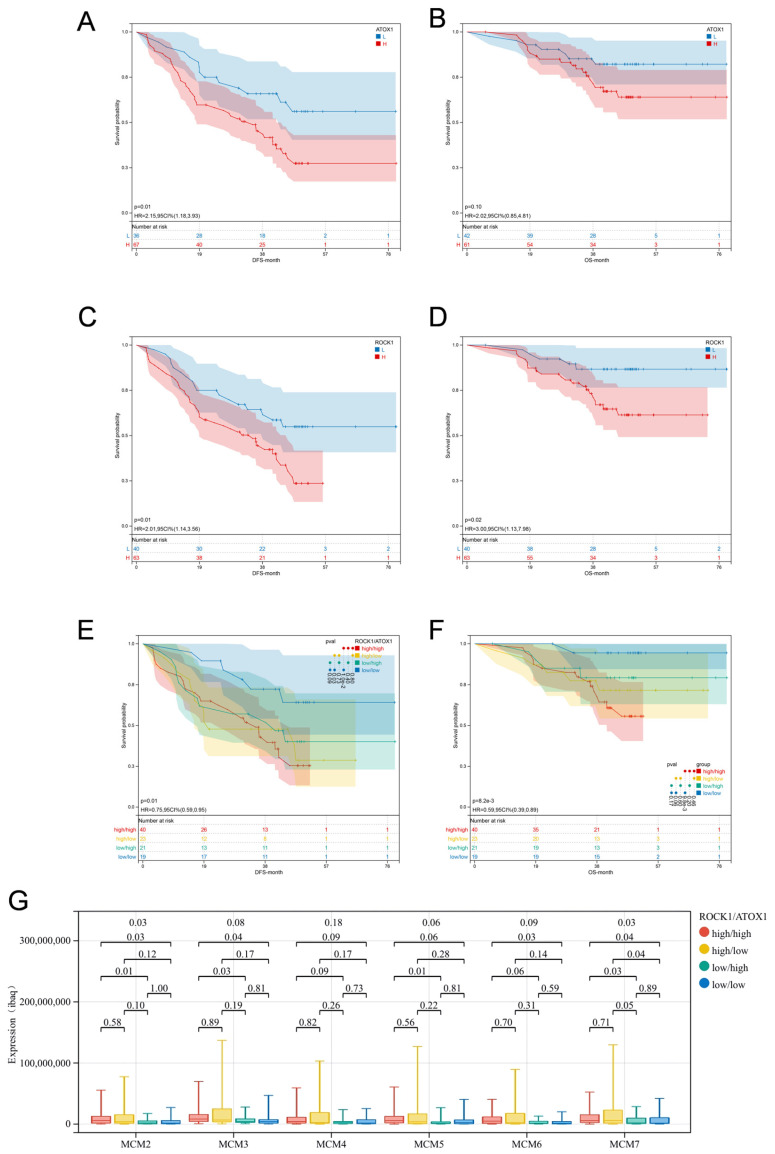
Association of ATOX1 and ROCK1 expression in lung adenocarcinoma (LUAD) with patient survival and MCM protein family expression. (**A**,**B**) High ATOX1 expression was associated with worse disease-free survival (DFS, *p* = 0.01) and overall survival (OS, *p* = 0.10) in patients. (**C**,**D**) ROCK1 high expression was associated with worse DFS (*p* = 0.01) and OS (*p* = 0.02). (**E**,**F**) Patients with dual-low expression of ATOX1 and ROCK1 had the best DFS (*p* = 0.01) and OS (*p* = 8.2 × 10^−3^). (**G**) Low expression of ROCK1 is associated with lower expression of MCMs, and is more pronounced at high ATOX1 expression.

This study re-analyzed the publicly available LUAD proteomic dataset generated in our prior investigation (Integrative Proteomic Characterization of Human Lung Adenocarcinoma, 2019), with the complete clinicopathological characteristics detailed in Supplementary Table S1. We found that high expression of ATOX1 was associated with both worse Disease-Free Survival (DFS) (log-rank test *p* = 0.01) and Overall Survival (OS) (log-rank test *p* = 0.10) (Figure 1A,B). Similarly, high expression of ROCK1 was also correlated with worse DFS (log-rank test *p* = 0.01) and OS (log-rank test *p* = 0.02) (Figure 1C,D).

Moreover, when considering ATOX1 and ROCK1 expression levels in combination, the ATOX1 low&ROCK1 low group exhibited the most favourable DFS (log-rank test *p* = 0.01) and OS (log-rank test *p* = 8.2 × 10^−3^) (Figure 1E,F). However, due to the limited sample size, Cox proportional hazards univariate and multivariate analyses were not performed. Subsequently, we investigated the correlation between the ATOX1/ROCK1 expression patterns and the expression of the MCM protein family. As depicted in Figure 1G, low ROCK1 expression was related to lower MCM expression, and this correlation was particularly strong under the condition of high ATOX1 expression.

### 3.2. Expression and Prognostic Value of ATOX1/ROCK1 Expression Pattern in Independent IHC Validation Cohort

**Figure 2:**
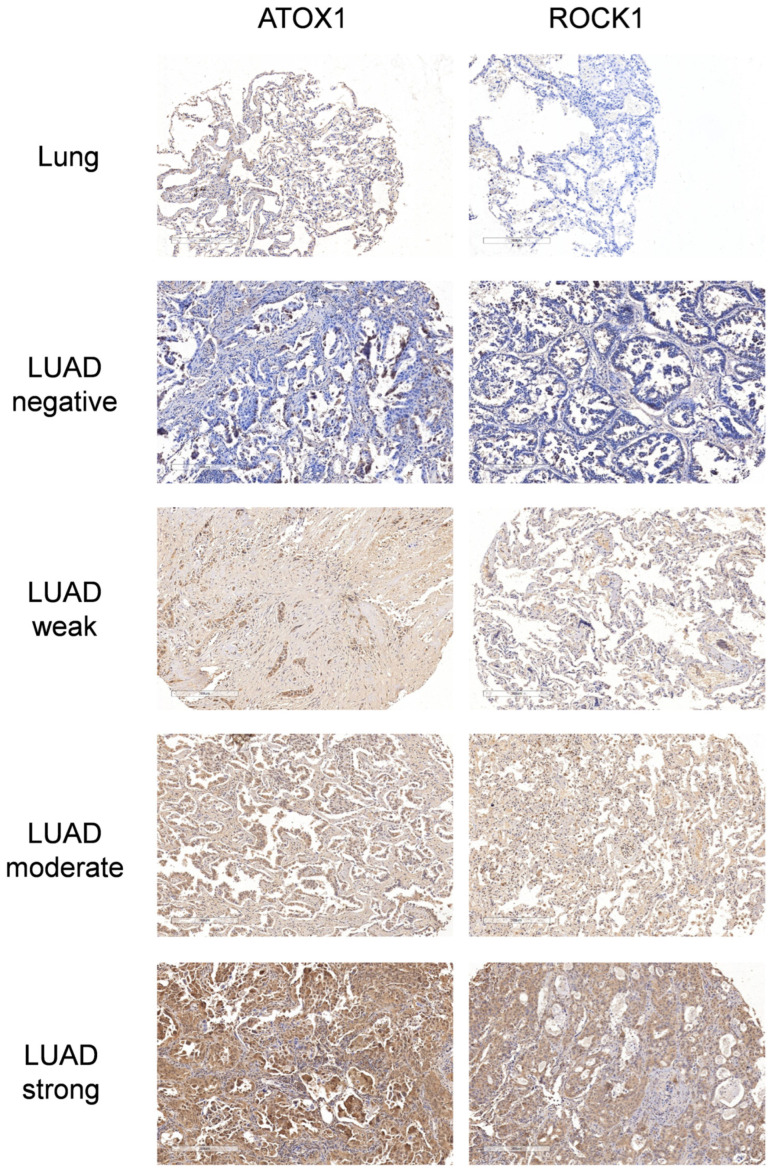
Immunohistochemical staining of ATOX1 and ROCK1 in lung adenocarcinoma (LUAD) and normal paraneoplastic lung tissue. This figure shows the immunohistochemical results of ATOX1 and ROCK1 in 35 LUAD patient samples and normal paraneoplastic lung tissues (NATs). There are four typical expression images, namely, negative, weakly positive, moderately positive, and strongly positive, in LUAD.

**Figure 3:**
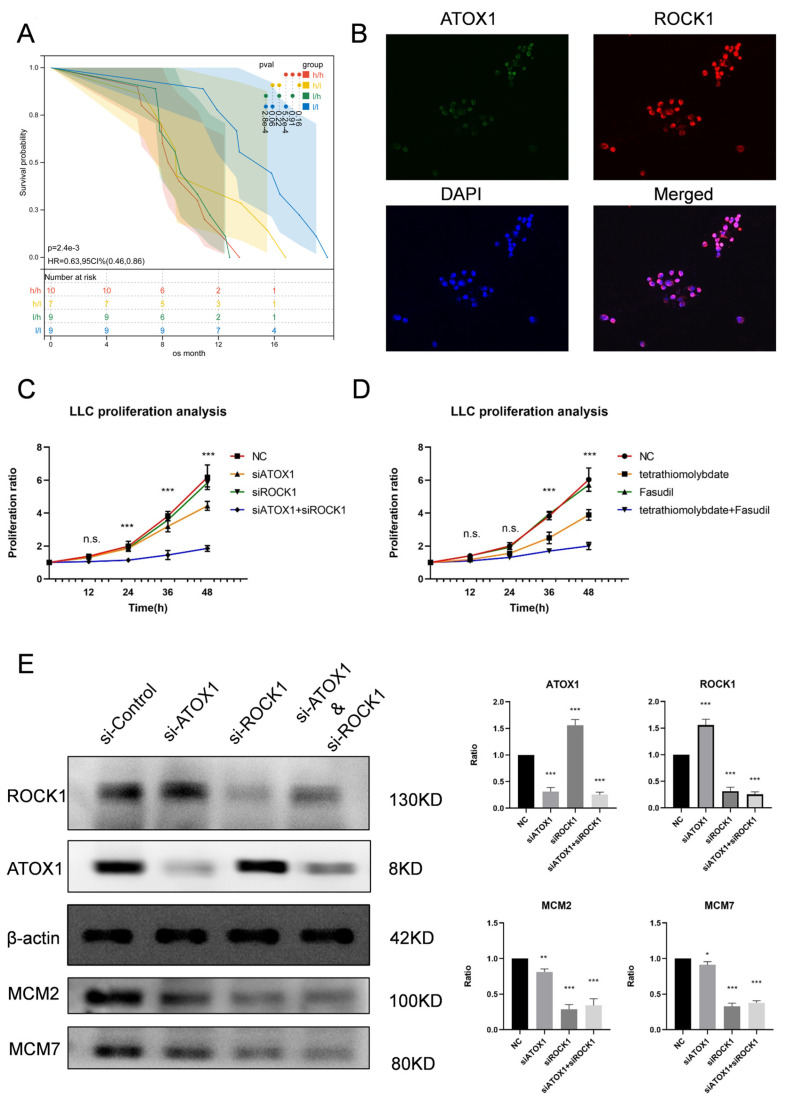
Effects of ATOX1 and ROCK1 expression on patient survival and LLCs. (**A**) Comparison of overall survival of patients with different ATOX1/ROCK1 expression patterns; the best survival was observed in the group with low ATOX1/ROCK1 expression (log-rank test, *p* = 2.4 × 10^−3^), and the expression of both was correlated with prognosis. (**B**) Immunofluorescence showed that ATOX1 and ROCK1 were highly expressed in LLCs and were localized in the cytoplasm. (**C**,**D**) Inhibition of ATOX1 alone (siATOX1: *p* = 1.34 × 10^−2^; tetrathiomolybdate: *p* = 1.38 × 10^−2^ vs. NC) or ROCK1 alone (siROCK1: *p* = 8.02 × 10^−1^; Fasudil: *p* = 4.89 × 10^−1^ vs. NC) did not significantly affect LLC proliferation by CCK-8, while co-inhibition profoundly suppressed proliferative capacity ((siRNA co-inhibition: *p* = 1.09 × 10^−4^; drug co-inhibition: *p* = 4.27 × 10^−5^, vs. NC, mixed-effects model). (**E**) In LLCs, ATOX1 and ROCK1 negatively feedback up-regulate each other and interfere with expression affecting MCM2/7 levels. Statistical symbols: n.s., not significant (*p* > 0.05); *, significant difference (*p* < 0.05); **, highly significant difference (*p* < 0.01); ***, extremely significant difference (*p* < 0.001).

In total, 35 patients with lung adenocarcinoma (LUAD) were included in this study, of whom 19 were male and 16 were female. The mean age of the patients was 59.0 ± 1.95 years; 26 patients had TNM stage III, and 9 patients had stage IV. At the end of the study, all patients had died, and the mean overall survival (OS) was 11.39 ± 0.63 months. Figure 2 illustrates typical immunohistochemical staining images of negative, weakly positive, moderately positive, and strongly positive expression of ATOX1 and ROCK1 in LUAD, whereas, in normal paracancerous lung tissues (NATs), both ATOX1 and ROCK1 were negatively expressed. Specifically, among the thirty-five samples, ROCK1 was expressed negatively in ten cases, weakly positive in eight cases, moderately positive in eight cases, and strongly positive in nine cases; ATOX1 was expressed negatively in eight cases, weakly positive in nine cases, moderately positive in ten cases, and strongly positive in nine cases. Overall, nine out of the thirty-five patients had low expression of both ATOX1 and ROCK1, and ten had high expression. Detailed information is shown in Supplementary Table S2. Finally, we compared the overall survival of patients with different ATOX1/ROCK1 expression patterns. In an unadjusted analysis, a statistically significant longer OS was observed in the ATOX1/ROCK1 low-expression group compared to the high-expression group (log-rank test, *p* = 2.4 × 10^−3^; Figure 3A). However, it is important to note that this analysis did not account for the potential confounding factors known to significantly impact LUAD prognosis and treatment response, such as EGFR mutation status, ALK fusion status, and specific therapeutic regimens. Therefore, while suggestive, this association requires validation in larger cohorts with rigorous multivariate analysis to determine the independent prognostic significance of ATOX1/ROCK1.

### 3.3. In Vitro Co-Inhibition of ATOX1 and ROCK1 Greatly Reduced LLC Proliferation

Both ATOX1 and ROCK1 were highly expressed and localized in the cytoplasm of Lewis lung cancer cells (LLCs), as confirmed by immunofluorescence (Figure 3B). We analyzed the proliferative capacity of LLCs when ATOX1/ROCK1 was inhibited by si-RNA interference or targeted drugs alone and in combination, respectively, using the CCK-8 assay. The results show that inhibition of ATOX1 alone did not affect LLC proliferation compared to NC controls at 48 h by mixed-effects model (siATOX1: *p* = 1.34 × 10^−2^; tetrathiomolybdate: *p* = 1.38 × 10^−2^), while ROCK1 inhibition alone also showed no significant effect at 48 h using the mixed-effects model (siROCK1: *p* = 8.02 × 10^−1^; Fasudil: *p* = 4.89 × 10^−1^). However, simultaneous inhibition of ATOX1 and ROCK1 significantly reduced the proliferative capacity of LLCs at 48 h (siRNA co-inhibition: *p* = 1.09 × 10^−4^, mixed-effects model; drug co-inhibition: *p* = 4.27 × 10^−5^, mixed-effects model) (Figure 3C,D).

### 3.4. ATOX1/ROCK1 Up-Regulated Each Other in a Negative Feedback Way

In Lewis lung cancer cells (LLCs), ATOX1 and ROCK1 up-regulate each other in a negative feedback manner. Interfering with ATOX1 expression using si-ATOX1 slightly decreased the expression level of MCM2/7, but resulted in the up-regulation of ROCK1 expression. Similarly, when si-ROCK1 was used to interfere with ROCK1 expression, its effect on MCM2/7 expression was more pronounced and also slightly induced ATOX1 expression (Figure 3E). This result suggests that a complex regulatory relationship exists between ATOX1 and ROCK1 in LLCs, and this negative feedback up-regulation mechanism may play an important role in cell physiology and pathology.

### 3.5. In Vitro, Co-Inhibition of ATOX1 and ROCK1 Inhibited LLC Migration

**Figure 4:**
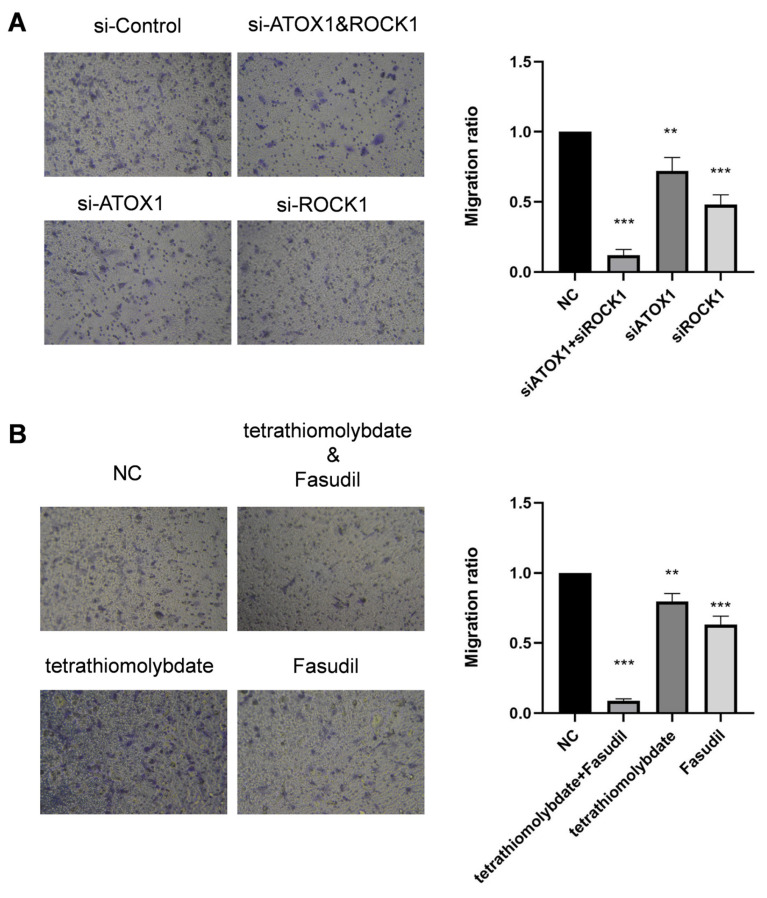
Inhibitory effects of ATOX1 and ROCK1 co-inhibition on LLC migration. (**A**). Transwell assay results for LLCs. Single treatments with si-ATOX1, si-ROCK1, tetrathiomolybdate, or Fasudil decreased cell migration. (**B**). Co-Co-inhibition of ATOX1 and ROCK1, either by combined si-RNAs or drugs, significantly enhanced the inhibition of LLC migration, showing the superiority of synergistic inhibition in regulating cell migration. Statistical symbols: **, highly significant difference (*p* < 0.01); ***, extremely significant difference (*p* < 0.001).

It was determined, using a Transwell assay, that either si-ATOX1 or si-ROCK1 alone decrease the migration ability of LLCs. Similar inhibitory effects were observed when LLCs were treated with tetrathiomolybdate or Fasudil, respectively. However, the inhibition of LLC migration was significantly enhanced when si-ATOX1 and si-ROCK1 were combined for co-inhibition or when tetrathiomolybdate was used in combination with Fasudil (Figure 4A,B). This indicates that the synergistic inhibition of ATOX1 and ROCK1 in regulating LLC migration had a stronger effect compared to individual inhibition, further revealing the key roles of ATOX1 and ROCK1 in the mechanism of LLC migration.

### 3.6. In Vivo Therapeutic Effect of Tetrathiomolybdate and Fasudil

**Figure 5:**
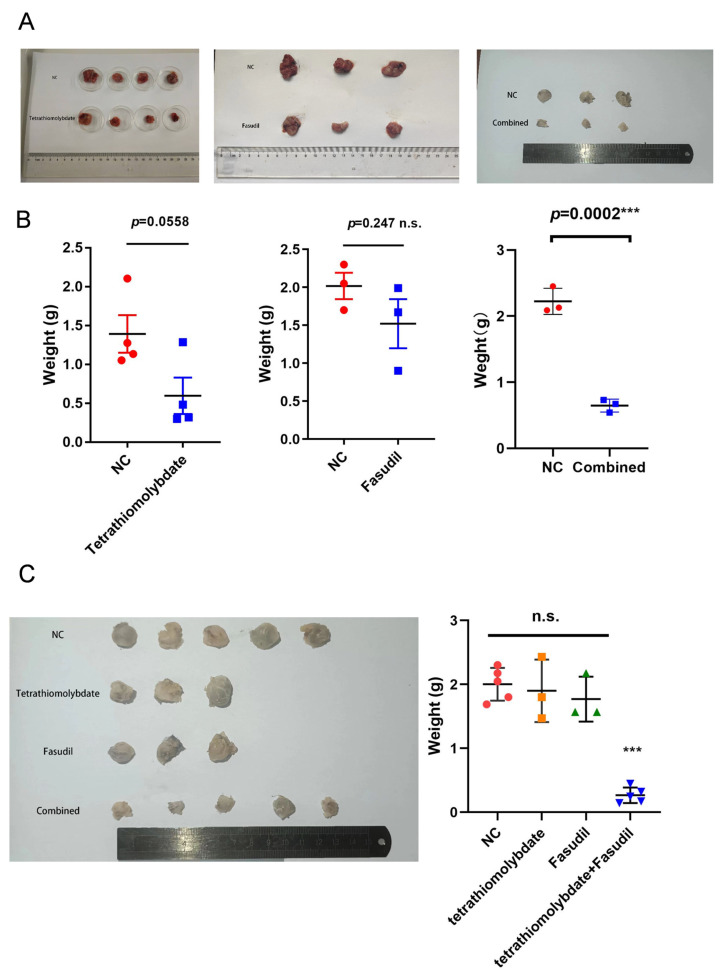
In vivo anti-tumour effects of tetrathiomolybdate and Fasudil. (**A**,**B**). Results from the LLC in vivo tumour model. Single-agent tetrathiomolybdate or Fasudil only showed a trend of inhibiting tumour growth (*p* = 0.0558, *p* = 0.247). Their combination significantly inhibited growth (*p* = 0.0002). (**C**). Data from the Mnu-induced tumour model. The combination therapy was more effective than single-drug treatment (*p* = 2.37 × 10^−5^). These show that the drug combination outperforms single agents in inhibiting tumour growth in vivo, providing a new strategy and basis for tumour therapy. Statistical symbols: n.s., not significant (*p* > 0.05); ***, extremely significant difference (*p* < 0.001).

In an in vivo tumour model of Lewis lung cancer cells (LLCs), either tetrathiomolybdate or Fasudil alone showed a tendency to inhibit tumour growth, but did not reach a statistically significant level (*p*-values of 0.0558 and 0.247, respectively). In contrast, tetrathiomolybdate significantly inhibited tumour growth when combined with Fasudil (*p* = 0.0002) (Figure 5A,B). Similarly, in the mnu-induced tumour model, the combination of tetrathiomolybdate and Fasudil achieved a more pronounced therapeutic effect compared to single-drug treatment (*p* = 2.37 × 10^−5^) (Figure 5C). This series of experimental results fully demonstrates that, in an in vivo setting, the combination of tetrathiomolybdate and Fasudil significantly outperforms the single-agent inhibition of tumour growth, providing a new combination strategy and theoretical basis for tumour therapy.

## 4. Discussion

In the current field of tumour therapy, LUAD, as a common tumour, has always been a major clinical challenge in the absence of effective treatments. Single-pathway inhibition is often difficult to improve patient prognosis, and combination therapeutic approaches have become an inevitable trend. The present study focused on the roles of ATOX1 and ROCK1 in LUAD and achieved a series of significant results.

ATOX1 is involved in regulating copper levels in tumour cells through binding and transport of copper ions. Copper is essential for cellular energy metabolism, signalling, antioxidant defence, etc. [22]. Tumour cells, due to their rapid growth and proliferation, require much more copper than normal cells [23], which makes it highly likely that ATOX1 will play a facilitating role in copper metabolism in tumour cells. Although the therapeutic strategies directly targeting ATOX1 are still immature, given the key position of copper metabolism in tumours, regulating ATOX1 is undoubtedly one of the feasible strategies to indirectly influence tumour progression. In particular, the recent discovery of the mechanism of ‘copper death’ (cuproptosis) has highlighted new opportunities for targeting copper homeostasis to induce cancer-specific death [24], providing a more robust theoretical basis for targeting copper metabolism through ATOX1.

ROCK1 is an important effector molecule in the RET and Ezrin signalling pathways, as it promotes migration and chemotaxis of lung adenocarcinoma cells through interaction with Ezrin under the regulation of the RET signalling pathway [25,26], which involves cytoskeletal remodelling and enhancement of cell motility, thereby promoting tumour metastasis [27]. However, there is a lack of previous animal and clinical studies on ROCK1, and the present study successfully fills this gap. ROCK1, as a key node regulating tumour invasion and metastasis, its inhibitors (such as Fasudil) have shown potential in inhibiting the metastasis of various solid tumours. The findings of this study further establish its position in the LUAD treatment target map.

The prognostic value of dual-low ATOX1/ROCK1 expression underscores its potential as a biomarker for stratifying high-risk LUAD patients. Importantly, our preclinical models validate the therapeutic superiority of co-targeting these proteins. While tetrathiomolybdate (a copper chelator) and Fasudil (a ROCK inhibitor) individually showed modest effects, their combination achieved significant tumour suppression in both LLCs and MNU-induced models (*p* = 2.37 × 10^−5^). This synergy is particularly compelling given the limited clinical success of copper-targeted therapies in solid tumours, suggesting that dual inhibition could circumvent resistance mechanisms linked to compensatory pathway activation. Furthermore, the correlation between ATOX1/ROCK1 and MCM2/7 expression implies that their inhibition may disrupt DNA replication machinery, adding another layer to their anti-proliferative effects. Given that the MCM complex is a key licencing factor for DNA replication initiation, and its overexpression in LUAD is often associated with poor prognosis and chemoresistance [28,29], this finding suggests the combination therapy might overcome resistance related to DNA replication processes.

While our study provides foundational insights, the limited cohort size (*n* = 35) constitutes a key constraint: although sufficient to detect significant prognostic stratification (*p* = 0.000), it fundamentally precludes reliable clinical prediction modelling due to inadequate statistical power for multivariable adjustments or predictive accuracy validation—specifically preventing meaningful subgroup analyses (e.g., stage-specific effects), adjustment for critical covariates (smoking status/molecular subtypes), and development of clinically applicable risk calculators, thereby necessitating validation in larger multi-centre datasets with expanded molecular subtype representation (e.g., EGFR-wildtype/KRAS-mutant) to establish predictive utility and precision stratification. Second, the exact molecular mediators of the ATOX1-ROCK1 feedback loop remain unclear; future studies should explore downstream effectors (e.g., MEK/ERK or LOX pathways) and epigenetic regulators [30]. Further investigation is especially needed into the specific molecular bridges underlying the complex interplay between cellular copper homeostasis and cytoskeletal motility and potential post-transcriptional regulatory mechanisms (e.g., non-coding RNAs) [31]. Third, the reliance on murine models may not fully recapitulate human tumour microenvironments. Translational efforts should prioritize humanized models or early-phase trials to assess safety and efficacy in patients. Patient-derived xenograft (PDX) models or murine models with humanized immune systems can be employed to better simulate the human immune microenvironment and therapeutic responses [32]. Critically, the combination therapy holds immediate promise for the subset of LUAD patients exhibiting dual-high ATOX1/ROCK1 expression (≈35% of cases, IHC H-score >200), where it may overcome compensatory resistance. Next-step clinical implementation requires initiating biomarker-driven Phase Ib trials in this subpopulation and developing companion diagnostics quantifying ATOX1/ROCK1 protein ratios.

## 5. Conclusions

In this study, based on publicly available proteomic data of lung adenocarcinoma (LUAD), we used bioinformatics analysis, in vitro cellular experiments, and in vivo animal experiments to deeply investigate the functions and potential therapeutic strategies of ATOX1 and ROCK1 in LUAD. It was found that high expression of ATOX1 and ROCK1 was associated with shorter disease-free survival (DFS) and overall survival (OS), and patients with double-low expression had the best DFS and OS, suggesting that they could be used as potential indicators for prognostic assessment. In vitro, ATOX1 and ROCK1 were highly expressed and localized in the cytoplasm of Lewis lung cancer cells (LLCs), and they negatively feedback up-regulated each other; inhibition alone had no effect on LLC proliferation, while combined inhibition significantly inhibited proliferation, and the inhibitory effect of combined inhibition or dosing on cell migration was stronger. In vivo experiments showed that the use of tetrathiomolybdate or Fasudil alone did not significantly inhibit tumour growth, while the combined use was effective. In conclusion, ATOX1 and ROCK1 are crucial in the development of LUAD, and their expression patterns can be used for prognostic assessment. Combined inhibition and drug administration provide a new strategy for the treatment of LUAD, but the relevant molecular mechanisms and the specific details of the combination therapy still need to be studied in depth.

## 6. Declaration

We declare that the experimental design, data collection, and analyses in this thesis on the role of ATOX1 and ROCK1 in LUAD treatment were performed by us independently. The experimental data are true and reliable, and have not been tampered with or falsified in any way.

The sources of the open-access proteomics data used in the study are clear, and the sources have been labelled in detail in this paper. All animal experiments were conducted in strict compliance with the relevant animal ethics regulations and guidelines to ensure that the welfare of experimental animals was safeguarded.

We confirm that none of the authors have conflicts of interest with any organization or institution that may influence the impartiality of this study. In the course of conducting this study and writing the paper, no undue financial support or other inducements for benefits were accepted.

There are no disputes over intellectual property rights in this research, and all opinions and conclusions are the original content of our research team, based on scientific experiments. All legal liabilities arising from the inaccuracy of the contents of this statement shall be borne by all authors.

## Data Availability

All data generated or analyzed during this study are included in this published article and its Supplementary Information Files. Anonymized clinical data are available from the corresponding author on reasonable request.

## Supplementary Materials

The following supporting information can be downloaded at: https://www.mdpi.com/article/10.3390/cancers17172887/s1, Table S1: Clinical, Genetic & Survival Data of 103 Cancer Patients; Table S2: ROCK1 & ATOX1 Data of 35 Cases.
